# Partly and Fully Supervised Physical Exercise Effects on Cognitive Functions and Movement Proficiency of Adolescents

**DOI:** 10.3390/ijerph192416480

**Published:** 2022-12-08

**Authors:** Aija Klavina, Viktors Veliks, Inta Gulevska, Aleksandrs Aniscenko, Juris Porozovs, Anna Zusa

**Affiliations:** 1 Department of Science, Latvian Academy of Sport Education, LV-1006 Riga, Latvia; 2 Department of Health Promotion and Rehabilitation, Lithuanian Sport University, 44221 Kaunas, Lithuania; 3 Department of Education, Psychology and Art, University of Latvia, LV-1586 Riga, Latvia

**Keywords:** physical exercise, cognitive functions, movement proficiency, problematic internet use, adolescents

## Abstract

This study explored the effects of partly supervised physical exercise program (PSPEP) intervention compared to fully supervised physical exercise program (FSPEP) on cognitive functions, movement proficiency and problematic internet use (PIU) in adolescents presenting combined unhealthy lifestyle behaviors. Method: Over a period of 16 weeks the PSPEP group (n = 14) engaged in strength, balance and flexibility exercises three times per week with one supervised session and two unsupervised. The FSPEP group (*n* = 13) practiced dance activities for 2 to 4 days per week including in training sessions the same exercises as for the PSPEP group. Prior and after the study adolescents completed the PIU scale, performed movement proficiency and cognitive function tests. Results: The PSPEP had significant effect on improvement of stress tolerance (*p* < 0.01, g = 1.08), while the FSPEP had significant effect on contributing general intelligence (*p* < 0.05, g = 0.90), color-word interference of the reading speed or color recognition (*p* < 0.01, g = 1.33), short- and long-term memory (*p* < 0.01, g = 1.72) and stress tolerance (*p* < 0.05, g = 1.06). The PSPEP had significant effect on improvement of the bilateral coordination (*p* < 0.01, g = 1.08). None of the two programs had effect on PIU. Conclusions: Engagement in PSPEP selectively contributed bilateral coordination and cognitive skills related to reaction to multiple stimuli. The FSPEP had multiple significant effects in improvement of cognitive outcomes.

## 1. Introduction

Physical activity is fundamental to overall development of adolescents and affects many aspects of their health [1,2]. Longitudinal research studies indicate that daily physical activities (PA) in adolescents are associated with significant short- and long-term health benefits in physical, emotional, social, and cognitive domains across the life span [3,4]. As such, it is important integrate physical activity into the daily lives of adolescents and provide foundation in facilitating and maintaining a healthy, active lifestyle throughout adulthood [5,6]. Sport and regular physical activities are essential part in European Union (EU) policy addressing health of children and adolescents [7]. Participation in sport activities is among the favorites of young people in Europe, indicating that over one third (35%) of young adults (15–29 years of age) participate in sports club activities [8]. However, the large-scale international studies have reported that only 15–20% of adolescents in Europe engage in daily physical activity according to the World Health Organization (WHO) guidelines (60 min per day of moderate to vigorous physical activities) [5,9]. 

In Latvia local municipalities provide out of school sport programs in accredited sport educational institutions, called sport schools. Currently there are 82 sport schools providing 41 sport programs (beginners to advanced level). About 25% of children and adolescent in Latvia (total 40 278 children and adolescents age from 5–25 years) participate in out of school sport programs [10]. However, the national survey outcomes from 2018 on school age children health in Latvia have shown that only 18.8% 11-, 13- and 15-years old students comply with PA guidelines of 60 min daily vigorous and moderate physical activity [11]. The contradiction between numbers of adolescents involved in organized sports and those who comply with international PA guidelines is obvious, while haven not been explored. 

Moreover, during the last three years COVID-19 pandemic periodically put millions of adolescents into the home environment for several weeks and months with limited possibilities to participate in organized physical activities with friends, classmates and teammates [12]. In Latvia during COVID-19 pandemic sport instructors and coaches continued to provide out of school PA programs according to social distancing regulations, for example, remote sport sessions or outdoor activities in small groups (up to 10–15 participants). It has been noted that social distancing increased vulnerability to mental health problems such as higher prevalence of stress, anxiety, or concentration and attention problem leading to poor academic achievements [13,14,15]. Also, recent evidence indicates that social restrictions during COVID-19 have significantly increased engagement in sedentary behavior in adolescents [13]. While sedentary behavior international guidelines recommend less than 2–3 h of recreational screen time per day for children and adolescence [3], more than half (67%) of children increased their leisure screen time by 1.39 h/day and adolescents by 0.91 h/day during COVID-19 pandemic [14]. Furthermore, recent studies reported associations between increased screen time and problematic internet use behaviors [15]. Problematic Internet use (PIU) has been defined as the incapacity to control an individual’s use of the Internet, which leads to adverse consequences in one’s daily life [16], for example, psychological, social, school, and/or work difficulties [17]. Jelenchick, Hawk, & Moreno (2016) [18] have developed screening instrument to determine PIU in pediatric population (adolescents and young adults) including three subscales, (1) emotional impairment, (2) social impairment, and (3) risky/impulsive internet use.

It has been acknowledged that reduced PA and prolonged sedentary behaviors increase physical health risks as loss of muscular and cardiorespiratory fitness, reduced motor competence [13]. Motor competence (MC) is a person’s ability to be proficient in a broad range of locomotor, balance, and manipulative skills [19,20]. The movement proficiency is associated with the development and performance of human movement in a range of fundamental motor skills (e.g., throwing, catching, running) [21] and forms the foundation for developing more complex movement patterns [22], which may lead to lifelong PA maintenance [4]. Also, PA benefits blood circulation and oxygen supply to the brain, an increase greater tolerance of stress and improves cognitive functions [23]. Although, the association between PA and physical and mental health has been explored [24,25], the relation between different modes (e.g., supervised vs. unsupervised) and environmental aspects (e.g., school vs. home) of daily physical activity and cognitive functions requires more research. Limited available research in therapy related online supervision mode of activities in young adults could improve cognitive control of attention, ability of organization and planning, and problem solving [26]. Moreover, online exercise program requires self-discipline of adolescents regarding planning their time, following instructions during online sessions, complying with the adherence level defined in the program. According to previous research self-discipline predicted academic achievements among adolescents, including performance on standardised cognitive function tests [27]. 

Unhealthy lifestyle behaviors are infrequently seen in isolation, and multiple lifestyle risk factors commonly present in the same person. However, the evidence is limited regarding the relationship between the multiple unhealthy lifestyle factors and its association with cognitive and physical health in adolescents. Furthermore, understanding the prevalence of multiple unhealthy lifestyle behaviors in adolescents is important to prevent the development of disease in adulthood [9,28]. 

Cognitive functions include memory, executive functions, attention and visual-spatial perception [29]. During last decade there is increasing interest in the effects of physical exercise on cognitive functioning in adolescents [30]. Multiple studies implementing supervised interventions monitored by physical education teachers [24], sport coaches or instructors [6,31], health care professionals [2,32] concluded that higher levels of physical exercise are associated with better cognitive functioning. While many of studies reported a significant association of PA with one outcome measure (e.g., memory), most authors concluded that many aspects of this relationship remain unclear, and that further research is needed to explore the nature of multiple benefits of PA interventions. 

The primary objective of this study was to determine the effects of partly supervised physical exercise program (PSPEP) intervention compared to fully supervised physical exercise program (FSPEP) on cognitive functions, movement proficiency and problematic internet use (PIU) in adolescents presenting combined unhealthy lifestyle behaviors. The comparative analysis of effects of the two PA programs in this study are essential for designing a future large-scale study with PA interventions for adolescents in different settings.

## 2. Materials and Methods

### 2.1. Study Design 

The research was multilocation parallel, controlled group study. In total 27 adolescents were recruited from the two general education settings to participate in this study. Adolescents ranging in age from 11–14 years were eligible to participate in this study if they met two of the three following inclusion criteria during the last month, (1) did not comply with WHO physical activity guidelines for children and adolescents (at least 60 min PA/per day); (2) spent more than 2 h/day by screen (not counting usage for school); (3) reported at least two of the eight health complaints (headache, stomach-ache, backache, dizziness, feeling depressed, irritability, feeling nervous and difficulties getting to sleep) in 2 or more days/per week (see Table 1). Given the time of this study during COVID-19 pandemic restrictions applied to all education activities, including sport sessions, only small groups of students from the same class or group were permitted (up to 10–15 participants) to participate together. 

All participants who were included in this study had the written permission of their parent or guardian to participate in this study. Adolescents were excluded from this study if they had medical or physical conditions affecting their participation in physical exercises, or participated in other research studies during the time of this project targeting physical activity. There were no restrictions regarding the sex, ethnicity, or socioeconomic status of any of the participants. Data were collected at each of the participating school sites. Participants were assessed one week before and one week after the last training session by the research team involving two physiotherapists and two researchers in psychology. All researchers had more than 20 years of experience working with children and adolescents and using the outcome measures. The study was implemented according to the latest version of the Declaration of Helsinki and the ethical approval was granted by the Institutional Ethics Committee from the primary author’s institution

### 2.2. Intervention

The intervention period was 16-week (January–May, 2022) duration. The partially supervised physical exercise program (PSPEP) consisted of 48 sessions conducted for 40 min three days each week with one supervised training session (every Monday) and two unsupervised exercise sessions using recorded video (Wednesday and Friday were suggested). The training activities aimed to improve the participant’s balance, strength, and flexibility. In total, for the PSPEP the research team selected 44 exercises divided in the four subsets: (1) core/trunk muscles (12 exercises), (2) strength exercises for legs and arms (12 exercises), (3) stretching exercises for shoulder and hip muscles (10 exercises), and (4) static balance exercises (10 exercises) (the complete description of the PSPEP exercises available upon request). The exercises were selected from evidence based resources adrressing strength (resistance), balance and flexibility [33,34]. 

Supervised exercise training sessions were organized in the school setting (sport gym) after school time. Each session consisted of a warm-up (5 min) including light intensity movements, for example, walking, slow running, rotation movements in shoulder and hip joints, leg swing. The main phase (30 min) included 3–4 exercises from each of the four exercise subsets. The session ended with 5-min cooldown (e.g., light stretching or relaxation exercises). The exercise routines with different intensity stages were compiled from the four given exercise subsets. In general, movement skill development exercises were performed using participants’ own body weight with no specific equipment needed to make possible exercises performance in the home environment. The intensity of the training was examined with the help of a modified Borg scale (10 points). Participants were asked to perform each exercise for 10–20 s (e.g., stretching) or 8–12 repetitions of perceived exertion of 5–6 (‘somewhat hard’ to ‘hard’). Altogether, 3 to 4 exercises from each subset were performed during the training session. Every week participants received slightly modified training exercises which they performed together with the physiotherapist during supervised training sessions. After onsite session each participant received WhatsApp message indicating which exercises they have to use during the independent sessions at home that week. The video recordings were available in the google drive in the five folders dividing the warm-up, balance, strength, flexibility and cool-down exercises The google drive was shared with project coordinator, physiotherapists and adolescents. Adolescents were free to select time when to do exercises at home, while suggested days were Wednesday and Friday. Also, the physiotherapist discussed with participants the independent performance of exercises (e.g., self-monitoring by using mirror, intensity etc.). Students sent photos or videos of their exercise performance at home. The communication about independent sessions was done through WhatsApp and phone communications, followed by feedback from participants during weekly onsite training sessions. The training attendance was documented by the physiotherapist on weekly bases. The intervention was considered as feasible if ≥90% of the participants were able to complete the intervention. 

In this study FSPEP participants attended dance activities two to four days of about 1 h to 1.5 h sessions per week with 30 min balance, strength, and flexibility exercises included in their training sessions. For FSPEP participants the same exercises were used as for the PSPEP group. The dance group was part of out of school activity programs offered for school age children and adolescents. Participants involved in this study had two to five years dancing experience. Dance sessions were supervised by physiotherapist who is also certified dance instructor with more than 15 years professional experience. 

Both physiotherapists were experienced with developing and delivering the physical exercise programs to adolescents. To consider the fidelity of the implementation of intervention, they developed the online checklist of exercises used on weekly bases. The checklist was tested by other physiotherapist in earlier pilot study as part of the project. This helped to follow the intervention delivery as planned and agree in making amendments, when necessary. 

Table 1 provides descriptive characteristics of both study groups. 

### 2.3. Instruments

*Problematic internet use.* Adolescents’ problematic internet use (PIU) was measured by the Problematic and Risky Internet Use Screening Scale (PRIUSS), a validated adolescent screening instrument [35]. The PRIUSS is an 18-item screening scale for identifying PIU with questions structured into the three subscales: (1) social impairment (6 items), (2) emotional impairment (5 items), and (3) risky/impulsive internet use (7 items). The PRIUSS response selections use a Likert scale with scores of 0 through 4, including answers “never” = 0, “rarely” = 1, “sometimes” = 2, “often” = 3, and “very often” = 4. A PRIUSS score ≥ 26 indicates that the adolescent is at high risk for PIU, and score from 15–25 indicates intermediate risk for PIU [35]. The Cronbach’s alphas for the three subscales used in the current study were: 0.89 (social PIU), and 0.82 (emotional impairment) and 0.79 (risky/impulsive internet use).

*Movement proficiency.* Movement proficiency was assessed by four subtests of the Bruininks-Oseretsky Test of Motor Proficiency II (BOT-2) [36]. The BOT-2 consists of eight subtests of which the four were used in this study: (1) bilateral coordination (7 items), (2) balance (9 items), (3) running and speed (5 items), and (4) strength (5 items). The total point scores of the four subtests were then calculated, and converted to scale scores, standard scores, percentiles, age-equivalents and descriptive categories for each subtest. The time needed to complete the test was 15 min. 

*Cognitive Function Tests.* The psychophysiological parameters of participants were assessed using Vienna test system (VTS) that is widely used in various fields of psychology and pedagogy. The VTS is one of the leading computerized psychophysiological assessment methods developed by Austrian scientists [37]. The VTS contains broad spectrum of cognitive tests that fits with Cattell-Horn-Carroll model (CHC) and represents different aspects of cognitive abilities and general intelligence [38]. Following VTS tests were used in this study: (1) Adaptive Matrices test (AMT) to assess general intelligence (logical reasoning) according the Rasch model [39], (2) Cognitron (COG) to assess concentration; (3) Determination test (DT) to assess stress tolerance, (4) Figural Memory test (FMT) to assess figural learning performance, and short-term and long-term memory, (5) Stroop test (STROOP) to measure the color-word interference of the reading speed or color recognition, and (6) Reaction time (RT) test to assess the ability to react under simple stimulus. Each Vienna test consisted of the three parts: (1) verbal instructions, (2) training and (3) test. In the instruction part the participant was introduced by the researcher with the testing environment and equipment used to perform the test. During training, the participant performed two to three task trials and was instructed on performance of the test. Once the participant understood the task requirements the testing proceeded. The testing procedure was organized with one participant at a time in a quiet and closed room with computers, where participants could focus on the task performance. The time of completion of the six VTS subtests was one hour and half including the two breaks of 5 min and 30 min required during the FMT subtest.

*Healthy lifestyle behaviors.* For healthy behaviour data collection the questionnaire included eight questions on the subjective health complaints, two question on the daily physical activity and one question on the use of screen time. This survey was part of the large scale research project protocol [40]. The responses reflected adolescents’ daily habits during last six months. For subjective health complaints the five-point scale ranging from 1 = “rarely or never” to 5 = “about every day” was applied. Subjective health complaints were divided as somatic complaints (headache, stomach ache, backache, dizziness) and psychological complaints (feeling sad, anxiety, nervousness, sleep difficulties). A multiple health complaints variable was identified if the participant reported two or more health complaints observed four or more complaints weekly. 

To assess daily physical activity, adolescents were asked to indicate the number of days and hours over the past week during which they were doing moderate-vigorous physical activities. 

To assess daily screen time use, adolescents were asked to report how many hours per day they spent using information technologies (IT) (eg, watching TV, playing games, chatting, emailing, messaging on Internet etc.) during the weekday and weekend. A mean of the responses was used as the measure of screen time. A cut off of 3 h per day was used to allow for time spent reporting various IT, and to keep the results comparable to a recent international comparison study [3,9].

### 2.4. Statistical Analyses

Data were analyzed using SPSS Version 28.0. (IBM Corp. Released 2021. IBM SPSS Statistics for Windows, Version 28.0. Armonk, NY: IBM Corp) The descriptive data were presented as means, standard deviations, medians with corresponding 95% CI. The *t*-test was used for within-subject comparison of the group characteristics and intervention outcomes obtained from the outcomes of PRIUSS survey, cognitive function tests and movement proficiency measurements. Cognitive test results were calculated using Vienna Tests raw scores output values. Mixed- model analyses of variance (ANOVAs) for the two within-subjects factors was applied: baseline vs. after intervention ×2 between-subjects factor; PSPEP group vs. FSPEP group, to determine the effects of the intervention on each of the test outcomes. The within group analyses included all measurements before and after the intervention. The standartised Hedge’s g for *t*-test, and η^2^ (eta squared) values for the ANOVAs was used as an index of the effect size. Simple main effects were determined following observation of any significant interaction effects. Values around 0.2, 0.5 and 0.8 were interpreted as weak, medium and strong effect sizes for the Hedge g and 0.01, 0.06 and 0.14 for η^2^, respectively [41,42]. All observed tests results were compared with statistical significance levels of *p* < 0.05. 

## 3. Results

The results given below presents pre-post intervention outcomes within each group.

*Cognitive function test outcomes.*Table 2 presents the results of cognitive function outcomes compared before and after the intervention. Overall, the FSPEP group presented significantly higher changes in cognitive function outcomes after the intervention (dancing activities) than the PSPEP group. There was significant increase in the AMT test outcomes for the intelligence index scores (*p* = 0.02, g = 0.90) with 95% CI of [−1.315, −0.084] and number of correct items (*p* = 0.02, g = 0.91) with 95% CI [−3.719, −0.256] for the FSPEP group. Also, the FSPEP group showed a significant improvement in reaction time (RT) of reading outcomes in Stroop test (*p* = 0.00, g = 1.33) with 95% CI of [−0.082, 0.228]. Furthermore, the FSPEP group significantly improved the total score of figural learning performance (*p* = 0.00, g = 1.72) with 95% CI of [−11.582, −4.278]. Also, this group showed significant improvement in Determination test regarding the number of reaction items (*p* = 0.012, g = 1.06) with 95% CI of [−61.53, −8.95] and reaction time (RT) (*p* = 0.039, g = 0.847) with 95% CI of [0.005, 0.156]. However, this group did not present significant changes in any of Cognitron test scores. For the PSPEP group significant changes after intervention was found only in Determination test scores regarding outcomes of presented correct items (*p* = 0.01, g = 0.96) with 95% CI of [−41.643, −5.072], the number of stimuli (*p* = 0.001, g = 1.08) with 95% CI of [−40.295, −7.135], the number of reaction items (*p* = 0.007, g = 1.08) with 95% CI of [−45.368, −7.918], and reaction time (RT) (*p* = 0.002, g = 1.27) with 95% CI of [0.032, 0.124]. Overall, the physical activity programs had selective contribution presenting small to high effect on different cognitive functions in both groups with Hedge’s g ranging from 0.12 to 1.27 for the PSPEP group, and from 0.04 to 1.72) for the FSPEP, respectively. 

*Movement proficiency test outcomes.*Table 3 presents the results of movement proficiency based on BOT-2 scores before and after the intervention. There were no changes in total BOT-2 and subtest scores for the FSPEP group after the intervention (*p* > 0.01). For the PSPEP group a significant change after intervention was found in bilateral coordination outcomes (*p* = 0.00, g = 1.08) with 95% CI of [−1.594, −0.264], while no significant change was in total BOT-2 and other subtests scores (*p* > 0.05). The PSPEP had higher average effect on motor proficiency (mean of PSPEP Hedge g = 0.54) than FSPEP (mean of FSPEP Hedge g = 0.25).

*Problematic internet use (PIU) outcomes.* Problematic internet use (PIU) results (see Table 4) did not present significant changes in both study groups indicating similar scores across the PRIUSS subscales at baseline and after intervention (*p* > 0.05). The Hedge g were low in both groups indicating low effect of physical activity programs on PIU of participants in this study. 

Additional study outcome analyses with two-way-mixed-model ANOVA reported statistically significant time x group effect for physical activity hours per week (F(1, 20) = 4.948, *p* = 0.038, η^2^ = 0.198). Also, there was significant group and time main effect, F(1, 20) = 12.635, *p* = 0.002. η^2^ = 0.387 and F(1, 20) = 7.792, *p* = 0.011. η^2^ = 0.28, respectively, significant group factor effect for BOT-2 (F(1, 20) = 6.497, *p* = 0.019. η2 = 0.245), and for physical activity during week-days (F(1, 20) = 8.791, *p* = 0.008. η^2^ = 0.305). 

## 4. Discussion

This study aimed to explore and compare the effect of PSPEP and FSPEP on cognitive functions, movement proficiency and problematic internet use outcomes in adolescents presenting unhealthy lifestyle behaviors during one months before the intervention. It should be noted that the assessment and intervention process was affected by social restriction during COVID-19 pandemic. While the education process in schools and out of school programs (e.g., in sport clubs and sport schools) in Latvia maintained onsite throughout the time of this study, occasionally some participants with positive diagnose of coronavirus were sent to quarantine and could not participate in supervised sessions for one or two weeks. Also, the group sizes in out of school programs were reduced to maximum 10–15 persons in one session to avoid close social contacts. These organizational changes affected the quality of training sessions (e.g., shorter sessions, less time to teach new skills). However, the adherence rate was acceptable 75–80% with no dropout in both groups during the study. 

Regarding the obtained results, it should be first stated that the PSPEP group after intervention reported increased hours spent in weekly physical activities. This was not surprising outcome, since adolescents of the PSPEP group during intervention were involved in PA sessions three times per week (supervised in the school and independent at home) that was not part of their daily life before this study. While earlier studies demonstrated that school-based physical activity interventions have been effective for reducing sedentary behavior in adolescents [43], this study presented promising intervention outcomes in home setting. 

However, this group did not present significant changes in overall cognitive functions after intervention. The only significant improvements was in subitems of the Determination test outcomes showing that adolescents improved ability to respond to multi-stimuli (visual and acoustic signals) at higher speed than before intervention. The cognitive mechanisms involved in this task are related to psychophysical stress that arises from the need to provide continuous, rapid reaction and different responses to rapidly changing stimuli. Also, earlier studies have demonstrated significant association between increased physical activities and improvements in reaction time and stress control [31,44]. Furthermore, the FSPEP group had significant improvements related to number of total test items and reaction time in the Determination test, while the number of correct items and stimuli did not change. The higher changes in reaction time tests PSPEP group outcomes after intervention might be related to effects of specific physical exercises selected for the PSPEP targeting strength in core/trunk muscles, legs, arms as well as stretching and static balance requiring concentration. Moreover, the unsupervised sessions required to focus attention on visual and verbal instructions provided in the video recordings. Previous study has demonstrated that exercise, both regular and irregular, improves the cognitive skills (e.g., executive functions) [45]. Furthermore, other authors suggest that structured physical activity (e.g., muscle strengthening and stretching and bone strengthening exercises) facilitates working memory by increasing the efficiency of evaluating the stimulus [46], while the literature lacks an explanation for such selective effects of physical exercise on cognitive functions. For example, Tomporowski et al. (2015) [47] concluded that during physical activity, cognition may or may not be challenged by performing unfamiliar and difficult movements. In this study the within group data outcomes showed that PSPEP intervention was effective in improving cognitive functions in adolescents. However, these results should be treated with caution since between group data comparisons were not addressed for primary study outcomes. Also, the FSPEP group presented significant changes in multiple cognitive function outcomes, for example, in general intelligence and logical reasoning (Adaptive Matrices test), reading scores (Stroop test) and as mentioned above, in reaction time (Determination test). It might be explained by participation of some participants in regular dance activities for long period of time (2 to 5 years). While dancing is considered aerobic activity, it requires movement coordination, balance and strength. According to earlier research different aerobic physical exercises might improve the structural connectivity of the frontal brain areas, mediating the positive effects of physical exercise on cognitive functions [30,48]. 

Furthermore, this study showed that movement skills development exercises significantly improved bilateral coordination in participants of the PSPEP group while total BOT-2 scores did not change. The selective improvements in movement coordination outcomes might be attributed to the requirement during supervised and unsupervised sessions to manage cognitive as well as movement skill demands during video demonstrations. Overall, these outcomes were contrary to findings in other studies since evidence suggests that both short-term (8–16 weeks) [44] and long term (>6 months) [6] physical exercise interventions to improve movement proficiency in adolescents were effective. Interesting finding was that participants of the FSPEP group also did not have significant changes in the total BOT-2 outcomes after this study. The explanation for this could be that structured sport specific physical activities practiced regularly for long period of time aims to improve participants’ sports-specific skills, for example, dancing performance. However, these activities do not address wide range of new motor tasks including coordination, movement quality aspects and control underlying a particular motor outcome [49].

The PRIUSS results assessing the problematic internet use did not change after intervention in both groups. These outcomes partially disagree with previous studies. For example, Suchert et al. (2015) [50] reported analyses of 11 studies concluding that excessive screen time use, including problematic internet use, is a risk factor for mental health, while there is weak evidence of effect for independent displacement of excessive screen time by physical activity. However, Rahimi-Rigi and colleagues (2019) [51] suggested that physical exercise-based intervention can be effective in prevention of mental health risks diseases, including internet addiction and problematic internet use. In recent meta-analyses authors concluded that exercise-based intervention can effectively reduce dysfunctions of autonomic nervous system and to some extent normalize the structure of specific parts of the central nervous system [32]. It should be noted that during COVID-19 pandemic adolescents experienced limited interactions with peers, increasing a gap on their social network that has been compensated by virtual interactions. This might explain the outcomes of PIU scores across the study groups. 

Additional analyses from two-way-mixed-model ANOVA demonstrated significant time x group effect for physical activity hours per week that was expected, as PSPEP group increased PA levels per week because of participation in intervention. Furthermore, the significant group and time effect for BOT-2 outcomes and weekdays in physical activity was expected, such that PSPEP group increased number of days with physical activities during intervention and improved the bilateral coordination skills. These outcomes were in line with previous studies reporting that physical activity interventions for adolescents with health risks contributed improvement of motor skills, for example, flexibility, strength [52], cardiorespiratory fitness [53], and overall daily physical activities [2]. 

The absence of evidence of strong effects of intervention suggests that the observed changes in cognitive functions and movement proficiency may not be directly attributable to the intervention itself. Other factors, for example, COVID 19 related restrictions and quarantine time periods might affected participants’ motivation and efforts to perform exercises independently. 

### Study Limitations

There were multiple limitations in this study that should be considered when interpreting the results. First, self-reported survey was used for the selection of participants. It should be noted that self-reported data are prone to bias [54]. This aspect together with single-blinded RCT study design, may explain some differences between groups before intervention. Second, the intensity of training sessions measured by modified Borg scale presents challenge of its own due to individual differences of feeling fatigue in adolescents. This might be related to the outcome that, although the weekly physical activity level increased for the PSPEP intervention group, the overall movement proficiency did not change. Also, the interventions implemented independently in the home environment can be missing the controlling aspect of exercise intensity, volumed and duration. In addition, the current study design did not allow to control several contextual factors (e.g., time of day or day when physical exercises were performed at home for PSPEP and in dance sessions for FSPEP group; the activities performed during dance sessions for FSPEP group; physical activities performed by all participants during their free time) that may influence study outcomes. 

Third limitation of this study is the small sample sizes in both groups that may limit the generalizability of our findings. The COVID-19 related restrictions addressing the Latvian education system regarding number of participants in school classes and out of school organized activity groups affected recruitment of more adolescents in this study. Future research studies would be suggested to involve larger number of participants. Fourth, the COVID-19 pandemic affected socialization of adolescents that might decrease motivation to follow the unsupervised activity sessions while being at home alone. 

## 5. Conclusions

This study presented novelty of implementing partially remotely provided physical exercise intervention including pre-recorded videos demonstrating physical exercises facilitating more independent forms of physical activities in adolescents. The 16-weeks partly supervised physical exercise program (PSPEP) intervention implemented during COVID-19 pandemic had no significant effect on overall movement proficiency, cognitive performance and problematic internet use outcomes in adolescents presenting unhealthy lifestyle behaviors. However, the obtained evidence in this study showed that PSPEP intervention selectively contributed bilateral coordination and reaction time to respond to multiple visual and acoustic stimuli. Additionally, this study demonstrated that regular physical activities such as dancing appeared to make broader range of significant effects than specially selected physical exercises to improve cognitive outcomes (e.g., reaction time, memory). So, future research should explore the optimal exercise-based interventions for adolescents implemented in multiple environments including social behavior and self-efficacy component. 

## Figures and Tables

**Table 1:** Participant descriptive characteristics.

	Groups	
	PSPEP	FSPEP
N (boys)	14 (4)	13 (6)
Age ^a^	12.4 (0.3)	11.1 (1.1)
Physical activity/hours per week ^b^	3 (1, 4)	5 (4, 6)
Physical activity/days per week ^b^	0.75 (0, 1)	2.5 (1, 4)
Hours screen/weekdays ^b^	3 (2, 5)	2 (1, 3)
Hours screen/weekends ^b^	4 (0.5, 6)	2 (1, 5)
Subjective health complaints/week ^b^	2 (1, 5)	3 (0, 7)

^a^ Mean (SD) ^b^ Median [95% CI for median].

**Table 2:** Cognitive function assessment outcomes.

	PSPEP				FSPEP			
	Baseline Mean (SD)	After Intervention Mean (SD)	*p*	Hedges’ g	Baseline Mean (SD)	After Intervention Mean (SD)	*p*	Hedges’ g
***AMT***								
Intelligence Index	−2.48 (0.66)	−2.22 (0.59)	0.29	0.64	−2.20 (0.75)	−1.50 (0.73)	0.02	0.90
Correct items	7.07 (2.01)	7.86 (1.83)	0.29	0.40	7.85 (2.19)	9.83 (1.99)	0.02	0.91
***Stroop***								
Reading interference	0.17 (0.14)	0.11 (0.12)	0.26	0.42	0.10 (0.08)	0.07 (0.05)	0.33	0.38
Median RT—reading	1.13 (0.27)	0.99 (0.16)	0.09	0.64	1.06 (0.07)	0.92 (0.11)	0.00	1.33
Naming interference	0.06 (0.08)	0.07 (0.09)	0.73	0.12	0.13 (0.12)	0.10 (0.09)	0.59	0.21
Median RT—naming	0.96 (0.26)	0.87 (0.10)	0.28	0.39	0.99 (0.19)	0.92 (0.18)	0.34	0.37
***COG***								
Sum correct reactions	53.08 (4.34)	55.64 (3.31)	0.09	0.64	55.31 (2.28)	56.83 (2.16)	0.10	0.66
Mean RT—correct reaction	2.01 (0.35)	1.93 (0.26)	0.50	0.25	2.21 (0.38)	2.14 (0.47)	0.68	0.16
**FMT**								
Delayed free reproduction I	6.71 (1.63)	7.07 (2.67)	0.67	0.15	7.92 (1.18)	8.0 (2.29)	0.91	0.04
Delayed free reproduction II	6.50 (2.68)	7.00 (2.57)	0.61	0.18	7.62 (1.19)	7.83 (2.29)	0.76	0.11
Learning sum	27.14 (9.93)	32.43 (7.88)	0.13	0.57	29.15 (5.47)	37.08 (2.96)	0.00	1.72
Recognition—hits	8.14 (0.94)	8.64 (0.63)	0.11	0.60	8.62 (0.65)	8.75 (0.45)	0.55	0.23
**DT**								
Correct items	211.5 (22.66)	234.86 (24.36)	0.014	0.96	211.85 (27.36)	228.58 (22.53)	0.110	0.64
Number of stimuli	234.64 (19.63)	258.36 (22.88)	0.001	1.08	233.08 (27.85)	252.92 (24.81)	0.074	0.72
Reactions items	230.36 (19.13)	257.00 (27.94)	0.007	1.08	233.92 (37.70)	269.17 (24.55)	0.012	1.06
Median RT	0.86 (0.052)	0.79 (0.06)	0.002	1.27	0.88 (0.10)	0.80 (0.08)	0.039	0.84
**RT6**								
Reaction speed RT6	306.07 (75.21)	315.29 (41.54)	0.69	0.14	291.08 (36.84)	303.92 (40.97)	0.41	0.31

**Table 3:** BOT-2 test results.

	PSPEP				FSPEP			
BOT-2 (Points)	Baseline Mean (SD)	After Intervention Mean (SD)	*p*	Hedges’ g	Baseline Mean (SD)	After Intervention Mean (SD)	*p*	Hedges’ g
BOT-2	120.6 (11.30)	126.5 (9.32)	0.14	0.54	133.1 (8.03)	133.8 (5.32)	0.82	0.09
BC *	22.86 (1.09)	23.79 (0.42)	0.00	1.08	23.7 (0.67)	23.60 (0.69)	0.74	0.13
Bal *	34.36 (3.41)	35.93 (2.12)	0.15	0.53	35.5 (1.26)	34.80 (0.78)	0.15	0.63
RS *	38.29 (4.37)	37.71 (4.04)	0.72	0.13	41.9 (3.98)	43.00 (3.71)	0.53	0.27
Strng *	27.14 (3.97)	29.07 (4.35)	0.23	0.44	32.00 (2.70)	32.40 (1.71)	0.69	0.16

* BC—bilateral coordination; Bal—balance, RSA—running and speed, Strng—strength.

**Table 4:** RPIUSS test results.

	PSPEP				FSPEP			
	Baseline Mean (SD)	After Intervention Mean (SD)	*p*	Hedges’ g	Baseline Mean (SD)	After Intervention Mean (SD)	*p*	Hedges’ g
PRIUSS(total)	19.92 (8.54)	19.88 (6.60)	0.99	0.00	14.11 (7.68)	15.80 (10.30)	0.73	0.18
Social Impairment	7.58 (3.39)	7.00 (2.26)	0.67	0.18	4.78 (1.78)	5.80 (2.28)	0.36	0.48
Emotional Impairment	4.25 (4.51)	5.50 (4.75)	0.56	0.26	3.33 (3.04)	3.20 (4.32)	0.94	0.03
Risky Impulsive Internet Use	8.08 (4.31)	7.38 (2.97)	0.69	0.17	6.00 (3.93)	6.80 (5.40)	0.75	0.16

## Data Availability

The data that support the findings of this study are available from the corresponding author upon reasonable request.
